# Inherent vs. Induced Loop Gain Abnormalities in Obstructive Sleep Apnea

**DOI:** 10.3389/fneur.2018.00896

**Published:** 2018-11-02

**Authors:** Naomi Deacon-Diaz, Atul Malhotra

**Affiliations:** Department of Medicine, Pulmonary and Critical Care Medicine, University of California, San Diego, San Diego, CA, United States

**Keywords:** loop gain, obstructive sleep apnea, chemoreflex control, neuroplasticity, functional residual capacity

## Abstract

Unstable ventilatory chemoreflex control, quantified as loop gain, is recognized as one of four key pathophysiological traits that contribute to cause obstructive sleep apnea (OSA). Novel treatments aimed at reducing loop gain are being investigated, with the intention that future OSA treatment may be tailored to the individual's specific cause of apnea. However, few studies have evaluated loop gain in OSA and non-OSA controls and those that have provide little evidence to support an inherent abnormality in either overall chemical loop gain in OSA patients vs. non-OSA controls, or its components (controller and plant gain). However, intermittent hypoxia may induce high controller gain through neuroplastic changes to chemoreflex control, and may also decrease plant gain via oxidative stress induced inflammation and reduced lung function. The inherent difficulties and limitations with loop gain measurements are discussed and areas where further research are required are highlighted, as only by understanding the mechanisms underlying OSA are new therapeutic approaches likely to emerge in OSA.

## Introduction

Obstructive sleep apnea (OSA) is a condition in which the upper airway either partially or completely obstructs during sleep. The repeated bouts of concomitant hypercapnia and hypoxia, surges in sympathetic neural activity and frequent arousals lead to a wide range of life-threatening comorbidities, including cardiovascular disease, metabolic disorder, depression, neurocognitive damage and increased mortality. OSA affects 24% of men and 9% of women in the US aged 30–60 years. As obesity is one of the key risk factors for developing OSA, the increasing obesity epidemic is estimated to increase OSA prevalence (1). Although several treatment options for OSA exist, issues of high cost, discomfort, invasiveness and poor efficacy cause many patients to discontinue long-term treatment (2). Thus, there is a need for new OSA treatments to reduce the growing public health burden.

In recent years it has been recognized that four quantifiable traits of upper airway anatomy and neuromuscular control contribute to variable degrees in each patient to cause OSA (3). New research has focused on treatments targeting each trait, with the intention that future OSA treatments may be tailored to the individual's pathology to increase both efficacy and adherence (4). One of the four traits is the stability of ventilatory chemoreflex control, called loop gain. Therefore, there is growing interest in treatments that may alter ventilatory chemoreflex control as a potential treatment for OSA (5, 6). However, it is currently uncertain whether ventilatory chemoreflex control, and therefore loop gain, is in fact consistently abnormal in OSA patients. It is also uncertain what physiological mechanisms may be responsible for increased loop gain in OSA, whether any abnormalities are inherent and therefore part of OSA pathogenesis, or whether abnormalities are induced due to OSA and are possibly reversible. This article aims to summarize the current knowledge on loop gain in OSA which may help guide the development of treatments intended to modulate ventilatory chemoreflex control in OSA.

## Ventilatory loop gain

Obstructive sleep apnea is a fairly unique condition in that it only occurs during sleep. The state dependent nature of OSA is due to the fact that during wakefulness ventilation is not only governed by metabolic chemoreflex control, but also by a conscious drive originating from supra-pontine brain centers which allow, to a degree, conscious override of the chemical control system. This conscious control is essential to be able to coordinate breathing with emotion and locomotion and breath hold while performing functions such as swallowing and swimming (7, 8). However, it also affords protection as conscious drive will maintain breathing even in the event where chemical drive to breathe may be very low (9). At sleep onset the wakefulness drive is lost and ventilation is primarily governed by the metabolic chemoreflex control system (8). During non-REM sleep, if arterial CO_2_ (PaCO_2_) decreases only 3-6 mmHg below eupnic levels the central chemoreceptor drive to breathe ceases and central apnea ensues, until PaCO_2_ rises high enough to reinitiate central ventilatory drive (10). Ventilatory drive not only determines the level of activity of the thoracic pump muscles, but also the upper airway dilator muscles (11). Therefore, although central apnea is not characteristic of OSA, instability in ventilatory chemoreflex control may promote OSA as the upper airway is susceptible to collapse when PaCO_2_, and therefore neural drive to the upper airway muscles, is low (12).

**Figure 1:**
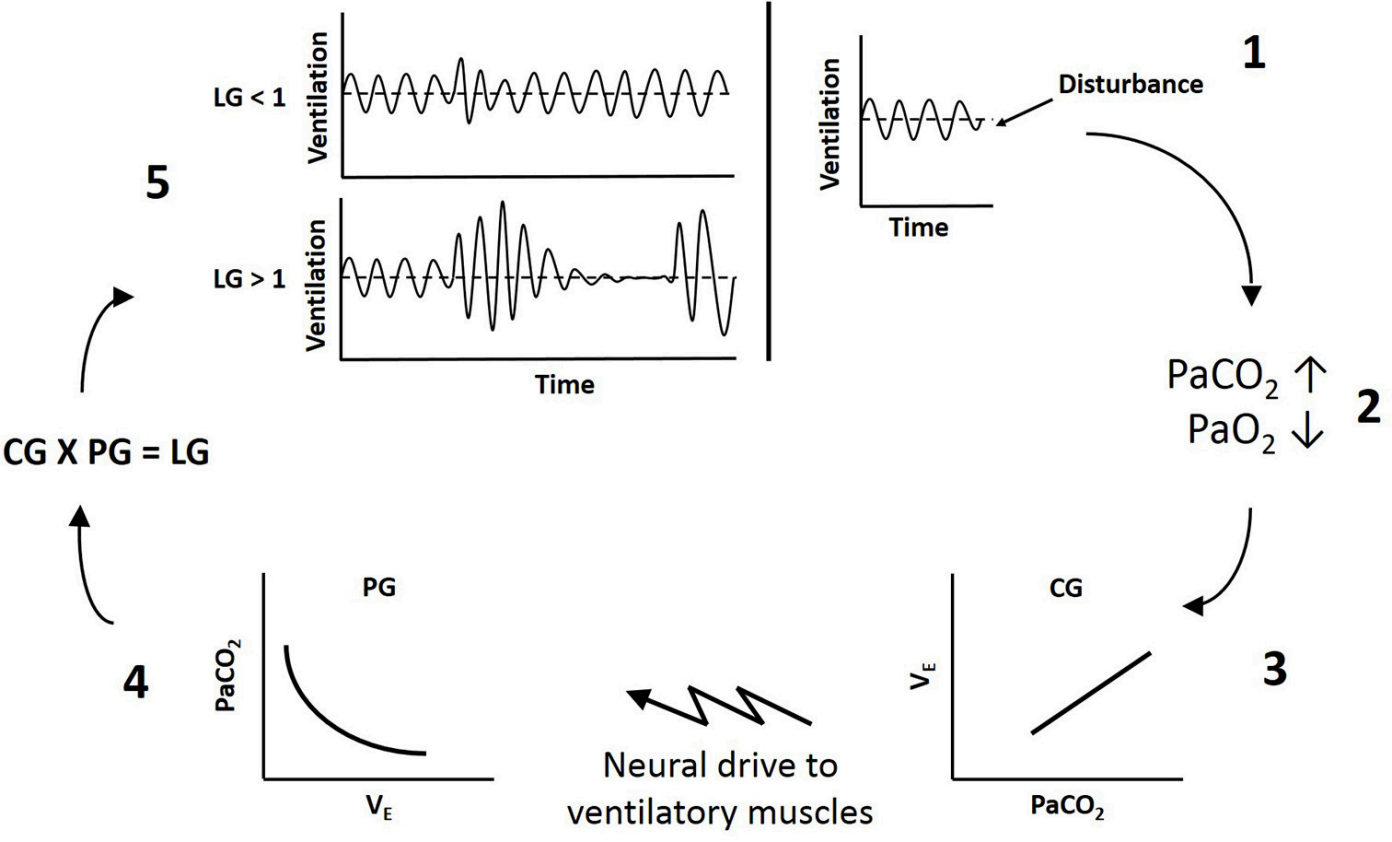
Schematic of ventilatory loop gain. **1**, A disturbance to breathing causes a reduction in ventilation below eupnea. **2**, Reduced ventilation increases arterial CO_2_ (PaCO_2_) and reduces arterial O_2_ (PaO_2_). **3**, Controller gain (CG) reflects the sensitivity of the peripheral and central chemoreceptors to blood gases and dictates the magnitude of neural drive to ventilatory muscles (ΔV_E_/ΔPaCO_2_). **4**, Plant gain (PG) represents the effectiveness of the lungs to change blood gases (ΔPaCO_2_/ΔV_E_). **5**, The product of controller and plant gain determines overall loop gain (LG). If loop gain is less than 1 (LG < 1), the fluctuations in ventilation will dampen out and breathing will stabilize. If loop gain is greater than 1 (LG > 1), the fluctuations in ventilation will increase in amplitude and instability will be self-perpetuating.

Loop gain is an engineering method used to measure the stability of the negative feedback chemoreflex control system. The overall loop gain of the ventilatory system reflects the ratio of the ventilatory response to the disturbance that elicited the response (LG = ventilatory response/ventilatory disturbance). Therefore, when breathing deviates from eupnea (the point where ventilation matches metabolic demand), such as during a hypopnea, if the ventilatory response that is elicited is equal to the disturbance (LG = 1), ventilation will correct blood gases to re-establish eupneic levels (Figure 1). If the ventilatory response is disproportionately larger than the disturbance (LG > 1), ventilation will not only correct the disturbance to blood gases, but will overshoot such that PaCO_2_ will be reduced below eupnic levels. The resulting hypocapnia will then induce hypoventilation, upper airway muscle hypotonia and a secondary airway obstruction (apnea or hypopnea depending on prevailing upper airway mechanics), such that respiratory events become self-perpetuating. Thus higher loop gain reflects less stable ventilatory chemoreflex control (13, 14).

Loop gain theory dictates there is a controller and a plant component, with a delay between the two (13). The controller senses a stimulus and dictates the output of the plant, which responds to decrease the stimulus. In ventilatory control, chemoreceptor sensitivity to blood gases reflects controller gain, and the effectiveness of the lungs to alter blood gases reflects plant gain. The product of controller and plant gain provides the overall loop gain of the system (LG = CG × PG) (14). Because there is a circulation delay between when ventilation begins to alter blood gases and when the chemoreceptors sense the change, if the gain of either the controller or the plant is too high, there is the potential for ventilatory overshoot producing instability in the system (13).

Carbon dioxide is considered the primary stimulus to chemoreflex control, because if PaCO_2_ is below a threshold level, hypoxic sensitivity is depressed and decreasing PaO_2_ will not alter ventilation (15). Therefore, controller gain reflects the slope of the ventilatory response to increasing CO_2_ (CG = ΔV_E_/ΔPaCO_2_) (12). Under hyperoxic conditions (albeit never naturally occurring) the peripheral chemoreceptors of the carotid bodies have diminished response to PaCO_2_. Only the central chemoreceptors in the brainstem will respond to increase ventilation, the gain of which describes central controller gain. During normoxia both the central and peripheral chemoreceptors contribute to the drive to breathe and the gain of the ventilatory response to CO_2_ will increase. Central chemoreceptors in the brainstem do not alter ventilation in response to changing PaO_2_. Only the peripheral chemoreceptors of the carotid bodies are sensitive to PaO_2_, increasing neural drive to ventilation in response to decreasing PaO_2_. If the additional stimuli of hypoxia is combined with hypercapnia, the contribution of the peripheral chemoreceptor drive to ventilation will increase and the gain of the ventilatory response to CO_2_ will increase further; a relationship described as hypoxic sensitization of the peripheral chemoreflex response to CO_2_ (16). Therefore, changes to either the central or peripheral chemoreceptor responses to CO_2_, or the peripheral chemoreceptor response to hypoxia, will alter controller gain. Chemoreflex sensitivity to both hypercapnia and hypoxia decrease progressively from wake to light NREM sleep to slow wave NREM sleep and are lowest in REM sleep (17, 18). Therefore, controller gain decreases with progressively deeper NREM sleep. REM sleep is characterized by tonic motor inhibition with bursts of phasic activity (19, 20). During the tonic phase ventilatory control is largely governed by the metabolic chemoreflex control system. However, the phasic phase provides additional stimulation to both inspiratory and expiratory muscles which somewhat overrides chemoreflex control and disorganizes breathing (19). In agreement with lower chemosensitivity in REM sleep than NREM sleep and additional neural drive to ventilatory muscles during REM sleep, loop gain in OSA patients is lowest in REM sleep and highest in lighter NREM sleep (21). Consequently, although breathing in REM sleep is characterized by an increase in respiratory events, breathing is typically more erratic without the underlying Cheyne-Stokes pattern characteristic of events driven by instability in chemical control (19, 20).

Plant gain quantifies the effectiveness of the lungs to alter blood gases, represented by the function of the isometabolic hyperbola (ΔPaCO_2_/ΔV_A_) (12). At rest ventilation is regulated to meet metabolic demand such that alveolar ventilation and PaCO_2_ are maintained relatively stable (i.e., eupnea). Due to the decreasing slope of the metabolic hyperbola during hypercapnia, plant gain increases as every unit increase in ventilation would produce a greater change in blood gases. Conversely, during hypocapnia the increasing slope of the metabolic hyperbola results in a reduction in plant gain, as every unit increase in ventilation would produce progressively smaller changes in blood gases (12). In people free of major cardiac or lung disease, it would be expected there would be no abnormalities in blood gas diffusion rates or circulation to affect plant gain. Therefore, in most OSA patients the main factor affecting plant gain is functional residual capacity. Reductions in lung stores of either CO_2_ or O_2_ will increase plant gain, because smaller tidal volumes will produce greater fluctuations in alveolar gas tensions. Thus reduced functional residual capacity increases plant gain (12, 13). Functional residual capacity decreases in the supine position (22), and further during sleep (23). Central obesity also decreases functional residual capacity due to increased abdominal compression (24). Therefore, obesity, particularly during supine sleep, would be expected to increase plant gain.

It is important to note that in OSA, the delay in the closed loop system is not just a function of lung to chemoreceptor circulatory delay. When the airway is obstructed, the delay is the duration of the event, as chemical drive continues to build and the ventilatory response cannot be expressed until the airway re-opens (25). Loop gain is also not a static value, rather it changes constantly. During an apneic event, as there is no ventilatory response, loop gain is actually zero (12). However, as chemical drive accumulates, because hypoxia sensitizes the carotid body hypercapnic response, progressive hypoxia and hypercapnia increases controller gain. As PaO_2_ is depleted and CO_2_ accumulates in the lungs, plant gain progressively increases such that when the airway opens, every unit increase in minute ventilation would produce greater fluctuations in alveolar gas tensions (12, 13). Thus, in OSA, the loop gain that is most relevant to apnea propagation is the loop gain at airway opening, when both controller and plant gains are at their peak. However, if the airway does not fully open the ventilatory response will be restricted, such that the net loop gain, being the loop gain that is actually expressed, will be lower than the chemical loop gain, being the actual drive to breathe (25). Most methods to quantify loop gain are performed during sleep with therapeutic continuous positive airway pressure (CPAP) to ensure the airway is fully dilated and there is no restriction to ventilation, thereby allowing quantification of chemical loop gain (26, 27). Upper airway mechanics are quantified within two other traits of OSA pathophysiology. The critical closing pressure is the airway pressure at which the passive airway collapses, and the upper airway recruitment threshold is the level of chemical drive required to activate the upper airway dilator muscles sufficiently to enable it to re-open after obstruction (28). Therefore, although upper airway mechanics will greatly influence net loop gain and therefore the loop gain contributing to event propagation in OSA, in patients free of cardiac and lung disease, the main factors that will affect chemical loop gain are factors that affect controller and plant gains.

## Evidence for pathophysiological role of unstable chemoreflex control in OSA

The pathophysiological role of unstable ventilatory chemoreflex control in promoting airway collapse in OSA due to hypocapnic hypotonia of the upper airways is well established. Obstructive apneas are followed by hyperventilation producing hypocapnia and respiratory depression (29). During spontaneous apneas, it is during the nadir of ventilation and upper airway dilator muscle activity, such as the genioglossus and alae nasi, that the airway collapses (30, 31). Imaging of the airway during spontaneous apneas has also shown the airway to collapse passively at end expiration (32). Even in healthy participants not normally susceptible to OSA, if hyperventilation and hypocapnia are induced during sleep, either with hypoxia or mechanical hyperventilation, participants develop periodic breathing and exhibit increased upper airway resistance, pharyngeal narrowing and obstruction during the nadir of ventilation (33–35). Additionally, in OSA patients, both hyperoxia to blunt the peripheral chemoreceptor response (i.e., reduce controller gain) and supplemental CO_2_ to prevent hyperventilation induced hypocapnic hypotonia of the upper airways, has been shown to stabilize ventilation and reduce the severity and frequency of apneic events (5, 29, 36).

## Is loop gain inherently high in OSA?

Although much evidence supports that unstable chemoreflex control contributes to propagating apneas in OSA, whether loop gain is elevated in OSA, and whether high controller or plant gain contribute to high loop gain, is currently unclear. Younes et al. found loop gain correlated with AHI (apnea-hypopnea index) in OSA patients (27), which some had extrapolated out to mean loop gain was higher in OSA patients vs. non-OSA participants. However subsequent studies have shown that the different traits of OSA pathogenesis contribute to varying degrees in different patients, such that it is possible for patients to exhibit a high AHI despite normal loop gain, and loop gain only correlated with AHI in patients with airways that collapse near atmospheric pressures (37, 38). Although these later findings highlight that loop gain is highly variable between patients and that loop gain is not the sole determinant of whether a patient has severe, mild or no OSA, as these studies did not include non-OSA control participants, they do not help to determine whether loop gain is elevated in OSA patients compared to people without OSA.

While several methods have been developed to quantify loop gain, each method yields vastly different results. For example, in two studies in OSA patients, loop gain has been reported as ranging from 0.33 to 0.42 when quantified using proportional assist ventilation to induce ventilatory instability (37), while a newer method employing CPAP drops has produced loop gain values ranging from 0.7 to 10.6 (28). Consequently it is not possible to make meaningful comparison of results from studies employing different techniques, and both OSA and control participants must be compared in the same study to determine if loop gain is elevated in OSA. However, likely due to limitations of currently available techniques to quantify loop gain (discussed below under Methodological limitations), few studies have actually compared loop gain between OSA patients and non-OSA controls.

We are aware of only nine published studies that have quantified loop gain in *both* OSA and controls. One study was conducted in elderly, in which OSA pathophysiology is known to be different from younger adults (39). Three were methods papers, two of which made no direct comparison between groups (3, 28). Of the six published studies that have compared loop gain between adult OSA and non-OSA controls (26, 38, 40–43), three found no difference in loop gain between groups (38, 41, 42). Hudgel et al. found loop gain was higher in OSA and concluded this finding was driven by higher plant gain in the OSA participants, with no difference in controller gain between groups (26). Gederi et al. found loop gain was higher in OSA due solely to higher controller gain, with no difference between groups in plant gain (40). Whereas Deacon et al. found loop gain was higher in OSA patients, but found no difference between groups in either controller or plant gains (43).

Although Gederi and colleagues reported loop gain was higher in OSA patients due to higher controller gain, the age, gender, height, weight and BMI of the participants was not reported (40). All of these factors influence both controller and plant gain. For example, obesity alone is known to increase sensitivity to CO_2_ (44). Thus, it is not possible to determine whether the higher controller gain exhibited in the OSA patients was associated with having OSA, or whether it was due to some other difference between the patients and control participants. Hudgel et al. used a pseudorandom binary CO_2_ stimulation test in which the ventilatory response to rapid perturbations in F_I_CO_2_ were used to compare ventilatory control in obese OSA patients and lean non-OSA control participants (26). By analyzing breath-by-breath variations in ventilation and CO_2_, this method allows quantification of both the controller gain component without plant gain feedback effects, called the open-loop response, and also the closed-loop response which incorporates both the controller and plant gain feedback. The authors did not report summary values of loop gain or its components, rather they compared the rate and magnitude of the ventilatory response to CO_2._ They found that the OSA patients had a greater peak and faster recovery in the closed-loop response, indicative of higher instability. However, there was no difference between patients and controls in the open-loop response (26). Therefore, loop gain was higher in the OSA patients due to higher plant gain. However, as the OSA patients were obese and the control participants were lean, and obesity is known to reduce functional residual volume which would be expected to increase plant gain, the authors concluded that less stable control in the closed-loop response in OSA patients may have been due to obesity, rather than being associated with OSA (26). Similarly, a more recent study by Sands et al. compared loop gain quantified using the CPAP drop method between obese OSA and both obese non-OSA and lean non-OSA (41). The authors reported that while there was no difference in loop gain between obese OSA patients and obese non-OSA control participants, loop gain was higher in both obese OSA patients and obese non-OSA controls compared to lean non-OSA controls. Although this method only quantifies the overall loop gain and controller and plant gain cannot be separately quantified, these findings also suggest high loop gain was associated with obesity dependent increased plant gain, rather than OSA (41). On the contrary, Deacon and colleagues rigorously matched control and OSA participants for gender, age, height and weight and found loop gain was higher in the OSA patients. Thus the findings of Deacon and colleagues are the first to support loop gain is higher in OSA patients independent of confounding variables such as obesity (43).

Of the published studies that have quantified loop gain in both OSA and controls, most have been conducted in patients that had been treated for several months to years with continuous positive airway pressure therapy (CPAP) (3, 38, 40, 41). Although two studies did not report treatment status, it is possible that patients in these studies were also treated with CPAP (26, 42). Therefore, the lack of strong evidence for high loop gain in OSA patients in these studies only suggests that loop gain is not *inherently* elevated in OSA patients. To determine whether loop gain is affected by treatment, Deacon and colleagues assessed newly diagnosed patients prior to commencing treatment and again following 2 and 6 weeks of CPAP treatment, and found no change in overall loop gain, or controller or plant gains, across the course of treatment. As controller and plant gains were not different between groups or affected by treatment, it is not possible to determine what mechanism contributed to the higher loop gain in the OSA patients in this study. However, the lack of treatment effect suggests loop gain may be inherently higher in OSA patients, or possibly that OSA induces increased loop gain through mechanisms which are not amenable to short-term treatment (43). As very few studies have compared loop gain between both OSA and controls, and the published data are inconclusive, it is necessary to evaluate the literature regarding the two major components of loop gain in OSA, being controller and plant gain.

## Intermittent hypoxia-induced high controller gain

Although data from loop gain studies are conflicting, there is strong evidence that when compared to well-matched controls, untreated OSA patients' exhibit abnormalities in chemoreflex control that increase controller gain. These abnormalities are treatment-reversible, suggesting high controller gain is not an inherent trait in OSA, but is induced by OSA itself. Importantly, the abnormalities seen in chemoreflex control of untreated OSA patients reflect neuroplastic changes that can be induced by intermittent hypoxia and which normalize following return to stable normoxia, strongly suggesting that high controller gain in OSA patients is induced by intermittent hypoxia. Supporting publications and key findings are summarized in Table 1 and discussed in detail below.

**Table 1:** Intermittent hypoxia-induced treatment-reversible high controller gain in OSA.

**OSA – DIFFERENCES TO HEALTHY CONTROLS**	**REFERENCES**
•Increased hypoxic sensitivity	(45)
•Normal hypercapnic sensitivity	(45, 46)
•Decreased eupneic P_ET_CO_2_ (LTF)	(47)
•Reduced CO_2_ reserve (LTF)	(47)
•Increased hypercapnic hypoxic ventilatory response	(48)
**OSA – REVERSAL WITH CPAP**	
•Hypoxic sensitivity decreases	(49)
•No change in hypercapnic sensitivity	(49)
•Eupneic P_ET_CO_2_ increases (loss of LTF)	(47)
•CO_2_ reserve increases (loss of LTF)	(47)
•Hypercapnic hypoxic ventilatory response decreases	(50)
**IH - INDUCES**	
•Increased hypoxic sensitivity	(51, 52)
•No change in hypercapnic sensitivity	(53, 54)
•LTF decreases eupneic P_ET_CO2	(51, 55)
•LTF decreases CO_2_ reserve	(55)
•Increased hypercapnic hypoxic response	(53)
**IH - NORMALIZATION FOLLOWING NORMOXIA**	
•Hypoxic sensitivity decreases	(54, 56)
•No change in hypercapnic sensitivity	(54)
•Eupneic P_ET_CO_2_ increases (loss of LTF)	(54, 56)
•Minute ventilation normalizes (loss of LTF)	(57)

*Key publications reporting the same abnormalities in chemoreflex control which increase controller gain in untreated OSA patients as can be induced in humans with intermittent hypoxia. In both OSA patients and following experimental intermittent hypoxia, re-exposure to stable normoxia results in normalization of chemoreflex control, supporting that intermittent hypoxia induces treatment-reversible high controller gain in untreated OSA patients*.

Chronic intermittent hypoxia in rats, designed to mimic OSA with 8 h daily exposures for several days to weeks, induces neuroplasticity at the carotid bodies which increases both basal neural discharge and hypoxic sensitivity (58, 59). In humans even acute intermittent hypoxia increases hypoxic sensitivity, while chronic exposure to intermittent hypoxia enhances this response (51, 52). In both humans and animals, hypoxic sensitivity returns to baseline following several days of re-exposure to stable normoxia (54, 56, 60). When compared to age and BMI matched non-OSA controls, OSA patients' exhibit elevated hypoxic sensitivity (45) and 1 month of continuous positive airway pressure treatment significantly reduces hypoxic sensitivity in previously treatment naïve patients (49). These findings support that carotid body hypoxic sensitivity is increased in untreated OSA patients due to intermittent hypoxia-induced neuroplasticity.

Acute intermittent hypoxia consisting of several short bursts can induce neuroplastic changes in cell bodies of ventilatory motor neurons such as those of the phrenic or hypoglossal nerves, which results in a persistent increase in neural activity for several hours after the last hypoxic stimulus, called long-term facilitation (LTF) (61). In intact animals, phrenic LTF increases ventilation for the same level of chemical stimulation (62). However, the increased ventilation is not due to increased sensitivity to CO_2_. Following intermittent hypoxia and the induction of ventilatory LTF in both animals and humans, chemoreflex tests have consistently shown there is no change in the sensitivity to hypercapnia (53, 54, 60, 63). Rather, when rats or humans are exposed to intermittent hypoxia to induce ventilatory LTF and then returned to a poikilocapnic environment, ventilatory feedback reduces P_ET_CO_2_ which then reduces minute ventilation, thereby constraining the expression of LTF (51, 53, 64). Thus, ventilatory LTF increases the magnitude of the ventilatory response to CO_2_ by causing a leftward shift along the metabolic hyperbola, without changing the sensitivity to CO_2_ above eupnea. As the apneic threshold in humans is not altered by intermittent hypoxia, ventilatory LTF reduces the CO_2_ reserve below eupnea, resulting in an increase in controller gain below eupnea (55). Like carotid body neuroplasticity, in both animals and humans, ventilatory LTF has also been shown to be reversible, with breathing and P_ET_CO_2_ returning to baseline levels several days after returning to stable normoxic breathing (54, 56, 57).

Data regarding hypercapnic ventilatory sensitivity in OSA patients have been conflicting, possibly due to lack of matching for obesity between groups, as obesity increases sensitivity to hypercapnia (44, 65), and inclusion of chronically hypercapnic patient in which CO_2_ sensitivity is depressed (66). Most studies report that untreated normocapnic OSA patients exhibit no difference in hypercapnic ventilatory sensitivity to well matched non-OSA controls (45, 46, 67, 68). This finding is in agreement with the lack of change in the sensitivity to CO_2_ following experimental exposure to intermittent hypoxia in both animals and humans (53, 54, 60, 63). However, whether OSA patients exhibit ventilatory LTF is uncertain. This may be because ventilatory LTF, as defined by an elevated minute ventilation, is usually experimentally determined with supplemental CO_2_ to maintain P_ET_CO_2_, thus preventing ventilatory feedback constraint of minute ventilation (51, 64). Although ventilatory LTF has not been reported as such in OSA patients, when compared to age, sex and BMI matched non-OSA participants, untreated OSA patients exhibit a reduced P_ET_CO_2_, reduced CO_2_ reserve and increased CO_2_ sensitivity below eupnea, which reflects the expression of ventilatory LTF and ventilatory feedback (47, 55). Following CPAP treatment P_ET_CO_2_ and the CO_2_ reserve increased in the OSA patients, further supporting the possible presence of ventilatory LTF in untreated OSA patients (47).

Intermittent hypoxia has also been shown to induce an increase in the ventilatory response of humans to combined hypercapnic hypoxia (53). In this study the authors reported LTF had not been induced, as minute ventilation was only elevated for the first 60–90s after the last hypoxic episode, then returned to baseline levels. However, P_ET_CO_2_ was reduced below baseline levels for the next 15 min of the room air breathing recovery period. This finding reflects the expression of LTF and ventilatory feedback constraint of ventilation observed in several other studies (51, 55, 64). During hyperoxia, there was no difference in the ventilatory sensitivity to hypercapnia post-intermittent hypoxia. As hypocapnia inhibits the peripheral chemoreceptor response to hypoxia, and intermittent hypoxia had induced a reduction in room air breathing P_ET_CO_2_ (due to LTF), the authors also found that there was no difference in the hypoxic ventilatory response when P_ET_CO_2_ was maintained. However, when CO_2_ was supplemented to raise P_ET_CO_2_ above the peripheral chemoreceptor threshold, hypoxic sensitivity was increased. Therefore, the findings of Mateika and colleagues reflects the combined effects of intermittent hypoxia-induced LTF and increased hypoxic sensitivity (53). Untreated OSA patients also exhibit an elevated ventilatory response to combined hypercapnic hypoxia, a finding which significantly attenuates following several months of CPAP treatment (48, 50). By definition, as the ventilatory response to chemostimulation (which incorporates both controller and plant gain) decreased following CPAP, these data show that loop gain decreased in OSA patients following CPAP treatment. Although non-OSA control participants were not included in these studies to determine whether the ventilatory response to hypercapnic hypoxia was higher in untreated OSA patients than non-OSA controls, a reduction in loop gain post-treatment supports that loop gain is increased in untreated OSA patients. Also, that elevated loop gain is not an inherent trait, rather it is induced by OSA itself and can be normalized with adequate treatment to prevent obstructive apneas.

Collectively, these data suggest that controller gain, and overall loop gain, could be elevated in untreated OSA patients due to the combined effects of intermittent hypoxia-induced ventilatory LTF and increased hypoxic sensitivity. As intermittent hypoxia-induced neuroplasticity is reversible, studies that have failed to find elevated loop gain in OSA patients may be because most [some authors did not provide treatment status (26, 42)] studies have evaluated OSA patients that had been treatment compliant for several months to years prior to evaluation (3, 38, 40, 41). Thus, any intermittent hypoxia-induced elevation of controller gain would have likely already been ameliorated. Although Deacon et al. found 6 weeks of CPAP treatment did not reduce elevated loop gain in previously treatment naïve patients, the lack of treatment effect may be due to limitations of the method used to quantify loop gain (43). The pseudorandom binary stimulation of CO_2_ method employed in this study only evaluates the chemoreflex loop gain to hypercapnia. Therefore, if patients did exhibit increased hypoxic sensitivity and increased sensitivity to CO_2_ below eupnea prior to treatment, and CPAP normalized these abnormalities, the method employed to quantify loop gain would not have evaluated these changes. Unfortunately, there are no loop gain methods currently available that separately quantify controller gain, which incorporate hypoxic sensitivity or CO_2_ sensitivity below eupnea.

## Obesity, lung volume and plant gain

Although loop gain studies support a role for obesity dependent reduced lung volume and increased plant gain in contributing to high loop gain in OSA, there are very little data comparing lung volumes or plant gain measurements between OSA and non-OSA controls matched for factors known to alter these variables. However, Deacon and colleagues found functional residual capacity and plant gain were not different between OSA patients and matched controls and CPAP treatment did not alter either measure. Although forced expiratory volume and forced vital capacity were only assessed at baseline prior to patients commencing CPAP treatment, so treatment effects could not be evaluated, both measures were significantly lower in the OSA patients (43). In a much larger study of 170 participants, Zerah-Lancner and colleagues compared spirometry, forced oscillation mechanics and gas exchange between moderate and severe OSA patients and non-OSA snorers, in which groups were matched for age, gender, BMI and smoking history. The authors also found no difference in total lung capacity, vital capacity, functional residual capacity or expiratory reserve volume between groups. However, lung function was impaired in OSA patients, as forced expiratory volumes and respiratory conductance decreased and respiratory resistance increased with increasing OSA severity (69). Gas exchange was also compromised in the OSA patients and correlated with OSA severity as PaO_2_ and O_2_ saturation negatively correlated with AHI and PaCO_2_ positively correlated with AHI (69).

These findings are in agreement with a growing body of work which supports that intermittent hypoxia experienced in OSA not only induces oxidative stress and inflammation systemically, but also within the lower airways, which may induce obstructive airways disease (70). This notion is supported by evidence that OSA commonly coexists with both asthma and COPD and has been shown to exacerbate disease progression in both conditions (70–72). CPAP treatment prevents the OSA associated decline in FEV1 in patients with comorbid asthma (72). In patients with comorbid COPD, CPAP improves FEV1 and reduces risk of exacerbations leading to hospitalization and also risk of increased mortality (71, 73). Exhaled nitric oxide (eNO) is a marker of respiratory inflammation and OSA patients exhibit elevated levels of eNO compared to non-OSA control participants. Also, eNO significantly increases in the morning after sleep in OSA patients but not in controls, the post-sleep increase in eNO levels correlates with AHI, nadir SaO_2_ and arousal index, and long-term CPAP treatment reduces eNO and markers of systemic oxidative stress (74, 75). Non-smoker OSA patients have also been shown to exhibit elevated levels of bronchial neutrophilia and IL-8 concentration in sputum compared to non-OSA participants. IL-8 concentration correlated with AHI and negatively correlated with nadir SaO_2_. One month of CPAP treatment did not significantly alter either marker of inflammation. However after 1 week of CPAP treatment airway hyperresponsiveness was increased and remained elevated at 4 weeks, which indicates that short duration CPAP treatment may aggravate airway inflammation, and that longer treatment duration may be required to observe improvements (76). Intermittent hypoxia has also been shown to induce oxidative stress, inflammation, altered immune responses, airway hyperresponsiveness and airflow limitation in rodents (77–80).

Although elevated plant gain may be acquired due to obesity dependent reduced lung volume, which may contribute to OSA pathogenesis and disease progression, the current data support that there is no inherent abnormality in lung volumes or plant gain in OSA. If OSA does reduce lung function, it would be expected that by compromising gas exchange, reduced lung function would decrease plant gain, not increase it. Therefore, OSA may induce a reduction in plant gain. However, due to the limited studies comparing plant gain, lung volumes and gas exchange between OSA and non-OSA participants matched for factors known to alter these variables, further research is needed to clarify the effects of obesity vs. OSA on plant gain.

## Methodological limitations

Discrepancies between the currently published studies comparing loop gain between OSA patients and non-OSA participants is likely due to the inherent difficulties and limitations with conducting this type of research. As obesity is one of the major causes of OSA, it is difficult to find control participants matched for BMI that do not have some OSA. It is also difficult to recruit OSA participants without commonly associated comorbidities that may affect loop gain, such as respiratory and cardiac disease. Similarly, while several methods have been developed to quantify loop gain, all have some limitations. Methods such as proportional assist ventilation, CPAP drops and cycle duty ratio of spontaneously occurring respiratory events essentially quantify the ratio of the ventilatory response to the disturbance, and therefore only provide the overall loop gain and cannot separate controller and plant components (27, 28, 81). Proportional assist ventilation produces hyperventilation to induce periodic breathing. However periodic breathing can only reliably be induced in severe OSA, therefore this method is not suitable for comparing loop gain between OSA and controls (27). Similarly, methods requiring spontaneous respiratory events cannot be used to calculate loop gain in non-OSA participants (82). To quantify controller and plant gains, it is necessary to analyze breath-to-breath variations in inspiratory and expiratory gases as well as ventilation. The pseudorandom binary stimulation of CO_2_ method does separately quantify controller and plant gains and can be conducted in both OSA and non-OSA participants (26). However, chemosensitivity, lung volumes and many other factors that affect loop gain change with sleep onset, and due to difficulty with maintaining sleep during chemostimulation and the fact that OSA patients would require CPAP during sleep to stabilize the airway, pseudorandom binary stimulation of CO_2_ is practically difficult to do during sleep. Methods have been developed that use spontaneous variations in ventilation and expiratory gases which allow for quantification of both controller and plant gain in both OSA and controls during sleep (40). However, to our knowledge there are no models in humans that have been published that incorporate O_2_ or oxyhemoglobin measurements. Therefore, the carotid body hypoxic contribution to controller gain is not evaluated. As intermittent hypoxia is known to increase hypoxic sensitivity and OSA patients exhibit treatment-reversible elevated hypoxic sensitivity, inability to quantify hypoxic sensitivity contribution to controller gain with currently available methods likely dramatically underestimates loop gain in untreated OSA patients.

## Summary

As several factors of upper airway anatomy and neuromuscular control interact to promote airway collapse, even a low/normal loop gain may be associated with pharyngeal airway collapse in participants with highly susceptible airways (38). Therefore, the lack of strong evidence for inherently high loop gain in OSA patients does not imply that loop gain does not contribute to OSA pathogenesis. Few studies have evaluated loop gain in both OSA and non-OSA controls, and data from studies that have provide conflicting results. Discrepancies between studies may be due to lack of matching for morphological traits known to alter both controller and plant gains, because most studies have not evaluated OSA patients both pre and post treatment, and due to limitations in all currently available methods to quantify loop gain.

Although loop gain studies are inconclusive, there is no evidence to suggest that lung volumes or plant gain are inherently different in OSA patients independent of other factors known to alter lung volume, such as obesity. There is also no evidence to suggest controller gain is inherently abnormal in OSA patients. However, intermittent hypoxia experienced in OSA may increase controller gain via neuroplasticity, and decrease plant gain via inflammation-induced compromised lung function. Although CPAP appears to ameliorate both abnormalities in chemoreflex control and lung function, whether total recovery is possible, or whether abnormalities persist with treatment, is unknown. Despite the inherent difficulties in conducting such research, future studies should aim to compare OSA patients both pre and post-treatment to well matched non-OSA participants, to properly assess treatment effects on controller, plant and overall loop gain. However, to thoroughly assess these aspects in both OSA and controls, development of new techniques that can separately quantify controller and plant gain contributions to overall loop gain, which can be conducted during sleep in both OSA and non-OSA participants, and which incorporate contributions of hypoxic sensitivity and CO_2_ sensitivity below eupnea to controller gain, will likely be necessary. Only by understanding the mechanism by which OSA appears to modulate both controller and plant gains, can treatments be developed which treat the cause of loop gain abnormalities in OSA.

## Author contributions

ND-D developed the topic and wrote the manuscript. AM helped structure the topic, edited the manuscript and approved the final draft.

### Conflict of interest statement

The authors declare that the research was conducted in the absence of any commercial or financial relationships that could be construed as a potential conflict of interest.
